# Mortality attributable to excess adiposity in England and Wales in 2003 and 2015: explorations with a spreadsheet implementation of the Comparative Risk Assessment methodology

**DOI:** 10.1186/1478-7954-7-11

**Published:** 2009-06-30

**Authors:** Christopher Kelly, Nora Pashayan, Sreetharan Munisamy, John W Powles

**Affiliations:** 1 University of Cambridge School of Clinical Medicine, Addenbrooke's Hospital, Hills Road, Cambridge, CB2 2SP, UK; 2 Department of Public Health and Primary Care, Institute of Public Health, University Forvie Site, Robinson Way, Cambridge, CB2 0SR, UK

## Abstract

**Background:**

Our aim was to estimate the burden of fatal disease attributable to excess adiposity in England and Wales in 2003 and 2015 and to explore the sensitivity of the estimates to the assumptions and methods used.

**Methods:**

A spreadsheet implementation of the World Health Organization's (WHO) Comparative Risk Assessment (CRA) methodology for continuously distributed exposures was used. For our base case, adiposity-related risks were assumed to be minimal with a mean (SD) BMI of 21 (1) Kg m^-2^. All cause mortality risks for 2015 were taken from the Government Actuary and alternative compositions by cause derived. Disease-specific relative risks by BMI were taken from the CRA project and varied in sensitivity analyses.

**Results:**

Under base case methods and assumptions for 2003, approximately 41,000 deaths and a loss of 1.05 years of life expectancy were attributed to excess adiposity. Seventy-seven percent of all diabetic deaths, 23% of all ischaemic heart disease deaths and 14% of all cerebrovascular disease deaths were attributed to excess adiposity. Predictions for 2015 were found to be more sensitive to assumptions about the future course of mortality risks for diabetes than to variation in the assumed trend in BMI. On less favourable assumptions the attributable loss of life expectancy in 2015 would rise modestly to 1.28 years.

**Conclusion:**

Excess adiposity appears to contribute materially but modestly to mortality risks in England and Wales and this contribution is likely to increase in the future. Uncertainty centres on future trends of associated diseases, especially diabetes. The robustness of these estimates is limited by the lack of control for correlated risks by stratification and by the empirical uncertainty surrounding the effects of prolonged excess adiposity beginning in adolescence.

## Background

The increase in adiposity over recent decades is widely believed to herald substantial adverse effects on health trends. Robust estimates of these effects are needed to inform societal responses to this challenge.

Recently, the UK Foresight Programme commissioned work to model possible future trends in obesity and their expected health effects [1]. The microsimulation programs developed for this project hold promise for exploring potential effects on population health through time. However, the published report provides only limited estimates of incident disease or death attributable to excess adiposity at the beginning of the 21^st ^century. (Graphical outputs are given for incident stroke, coronary heart disease, diabetes and arthritis attributable to BMIs in excess of 25 (figures 27 to 34) [1]). A summary measure of fatal effects – reductions in life expectancy at birth – is provided only for projected *increases *in adiposity *beyond *the levels prevailing in 2000–2004. The program is now publicly available Tim.Marsh@heartforum.org.uk but its "user-friendliness" remains to be confirmed.

Here we pursue a complementary path to exploring mortality attributable to excess adiposity in England and Wales in 2003. We also derive estimates for 2015, under varying assumptions. We use relatively straightforward cell-based computations in Excel and the model is provided as an online appendix.

Levels of adiposity are most commonly measured indirectly by the Body Mass Index (BMI). In estimating disease burdens attributable to excess adiposity, BMI has typically been treated as a categorical exposure (employing categories such as underweight, normal weight, overweight and obese). The method developed by the global Comparative Risk Assessment (CRA) project [2] compares aggregated mortality risks under actual or projected BMI distributions with the corresponding risks under a "theoretical minimum risk" counterfactual distribution of BMI. The CRA method respects the continuous nature of BMI distributions in contrast to methods that treat this exposure as categorical.

## Methods

We estimate mortality attributable to excess adiposity using Population Attributable Fractions. Conceptually, the Population Attributable Fraction (PAF) is the fraction by which the occurrence of a disease of interest would be reduced under a sustained alternative, more favourable, exposure distribution. For assessing the full effects of a given distribution of "exposure" (BMI), the appropriate comparator (or counterfactual) is a distribution deemed likely to confer "theoretical minimum risk". For our base case, we have followed the CRA project in using as a counterfactual a BMI distribution with a mean of 21 Kg m^-2 ^and a standard deviation of 1 Kg m^-2^, but this choice can be varied easily within the model.

Further details on the CRA methodology employed and its implementation in Excel are given in Additional file 1 and Additional file 2 and from the methodological [3] and substantive [2] publications of the CRA. (We also provide the full model with all the associated worksheets as Additional file 3).

In summary,



These aggregates of population risk can be thought of as proportional to "areas under the curve", made up, in a discrete approximation, of small strata of proportions exposed at a given level times the risk at that level, so that



where p_1 _refers to the factual (or predicted) and p_2 _to the counterfactual BMI distribution. In the CRA method, the RRs for all positions on the counterfactual exposure distribution are set to 1 and given that the sum of the probability distribution is 1, the formula simplifies to:



A discrete approximation to a normal distribution is implemented in Excel using the NORMDIST function. This enables calculation of the "distance travelled" on the X (BMI) axis in moving from a given position in the factual distribution to the corresponding position on the counterfactual distribution. This quantity times the slope of logRR on BMI gives the change of logRR and the exponent of this provides the RR_i _for each stratum of interest. The products of these RR_i_'s with the proportions exposed at each level can then be summed across the strata to give the PAFs, from the formula above. Potential shortcomings with this approach are considered further in the discussion.

We considered only attributable loss of life from deaths assigned (by the UK Office for National Statistics (ONS)) to colorectal cancer, breast cancer, cancer of the body of the uterus, diabetes, hypertensive heart disease, ischaemic heart disease and cerebrovascular disease – and these losses are expressed in the metrics of deaths, years of life lost (YLL, under varying weighting assumptions) and loss of life expectancy.

### Data sources

Distributions of BMI for 1997 to 2004 were obtained from the Health Survey for England (HSE) [4]. BMI trends in this period were projected forward, separately by age and sex groups to 2015. The "Mainsetup" sheet of the Excel model allows alternative projected trends in BMI to be selected. We have explored 3 main alternatives:

i. No change;

ii. A slowing rise – BMI increases by 30% less than the preceding linear trend;

iii. Extrapolation of the trend for 1997 to 2004.

Mortality and population data are from the Office for National Statistics [5,6]. Predicted all cause mortality rates for 2015 (by age and sex) are from the Government Actuary [7]. Two variants of the composition of risks within this mortality "envelope" are explored:

i. The composition remains constant from 2003–5;

ii. WHO predictions for high income countries are applied [8];

The CRA method requires as inputs observed associations between BMI levels and mortality risks for the causes of interest. We have taken these (in the form of slopes of logRR on BMI) from the meta-analyses performed for the CRA project [9]. No deaths under 30 have been attributed to excess adiposity.

### Estimation of attributable mortality

We considered only attributable loss of life from deaths assigned (by the UK Office for National Statistics (ONS)) to colorectal cancer, breast cancer, cancer of the body of the uterus, diabetes, hypertensive heart disease, ischaemic heart disease and cerebrovascular disease – and these losses are expressed in the metrics of deaths, years of life lost (YLL, under varying weighting assumptions) and loss of life expectancy.

The years of life lost as a result of death at a given age can be assigned on varying assumptions. We have incorporated 3 alternative user-selectable systems for deriving YLL weights:

i. The reference life tables used in the Global Burden of Disease and CRA projects. These have a life expectancy at birth of 80 years for males and 82.5 years for females;

ii. UK period life tables. Use of these would assign (as YLL) the expected number of years of life remaining under the current (period) UK life table at the age of death;

iii. The absolute time remaining to age 75, otherwise known as 'Person Years of Life Lost to Age 75 (PYLL75) – a metric used by the UK Office for National Statistics.

The streams of life lost under each of these systems may, optionally, be discounted at 3% per year and/or age-weighted (using the GBD age weights). We have followed the GBD convention in using both discounting and age-weighting [10] for the YLL estimations presented here. The setup page of the workbook provides a graphical display of the YLL weighting system chosen by the user selected inputs.

The attributable loss of life expectancy is calculated by subtracting mortality risks attributable to higher than optimal BMI from the overall mortality rates, using established methods [11].

## Results

### BMI distributions

In 2003, the mean BMI in England and Wales for persons 30 or older was 27.7 Kg m^-2 ^for males and 27.3 Kg m^-2 ^for females. If BMI levels increase linearly, they would rise by 2015 to 29.1 Kg m^-2 ^and 28.4 Kg m^-2 ^for males and females respectively (assuming constant age structure).

### Attributable mortality in 2003

**Figure 1:**
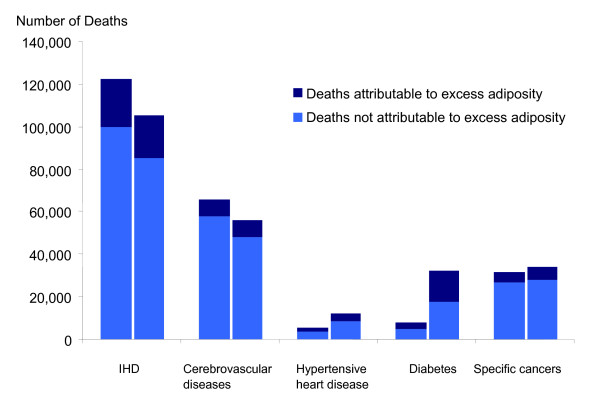
**Deaths from 5 causes of interest, by whether attributable to excess adiposity, England and Wales, 2003 (left columns) and 2015 (right columns)**. All cause mortality levels in 2015 as predicted by the Government Actuary, composition of mortality risks as predicted by WHO for high-income countries.

In 2003 the estimated attributable reduction in life expectancy was 1.05 years. There were approximately 41,000 attributable deaths. The years of life lost per attributable death average 12.4 using a period life table for England and Wales without discounting or age-weighting the streams of lost life. Using the GBD YLL metric (reference life table, with discounting and age-weighting) this was reduced to 6.4 years (Table 1). Males accounted for 52% of attributable deaths and 60% of YLL. The leading causes of attributable deaths were ischaemic heart disease (55%), cerebrovascular disease (20%) and diabetes (9%) (Figure 1).

**Table 1:** Mortality attributable to excess adiposity: deaths, YLL, PYLL75 and loss of life expectancy – total population, males and females, England and Wales, 2003 and 2015.

	**2003**	**2015 – composition of mortality risks same as in 2003–05**			**2015 – composition of mortality risks from WHO prediction for high-income countries**		
		
		**No change in BMI**	**BMI increase 30% below recent trend**	**BMI increase continues recent trend**	**No change in BMI**	**BMI increase 30% below recent trend**	**BMI increase continues recent trend**
**Total population**							
Attributable deaths	40,874	40,613	44,914	46,540	46,703	51,140	52,798
Years of life lost (YLL*)	261,153	254,862	275,618	283,591	280,594	300,364	307,941
YLL* per attributable death	6.39	6.28	6.14	6.09	6.01	5.87	5.83
PYLL75	134,805	136,866	147,023	150,615	145,220	154,202	157,337
Attributable loss of life expectancy (years)	1.05	1.04	1.13	1.16	1.16	1.25	1.28
							
**Males**							

Attributable deaths	21,250	20,072	22,869	23,897	22,133	24,937	25,944
Years of life lost (YLL*)	155,519	144,071	158,015	163,318	146,431	159,155	163,949
YLL* per attributable death	7.32	7.18	6.91	6.83	6.62	6.38	6.32
PYLL75	92,008	87,223	94,655	97,386	84,761	91,159	163,949
Attributable loss of life expectancy (years)	1.19	1.12	1.24	1.28	1.18	1.30	1.34
							
**Females**							

Attributable deaths	19,625	20,541	22,044	22,643	24,570	26,203	26,854
Years of life lost (YLL*)	105,634	110,791	117,603	120,273	134,163	141,209	143,992
YLL* per attributable death	5.38	5.39	5.33	5.31	5.46	5.39	5.36
PYLL75	42,797	49,642	52,369	53,229	60,459	63,043	63,839
Attributable loss of life expectancy (years)	0.92	0.96	1.02	1.04	1.14	1.20	1.23

* YLL weights use GBD reference life tables, 3% discounting and age-weighting.

All cause mortality levels in 2015 from Government Actuary's predictions, with composition of mortality risks assumed constant or as predicted by WHO for high-income countries.

The 41,000 deaths attributed to higher than optimal BMI in 2003 amounted to around 9% of all deaths in England and Wales. This included 77% of all diabetic deaths, 23% of all ischaemic heart disease deaths and 14% of all cerebrovascular disease deaths.

### Projections for 2015

The effect of varying assumptions about the future trend of BMI turned out to be surprisingly modest: increasing the attributable loss of life expectancy in 2015 from 1.04 years for both sexes combined in the case of no further increase in BMI to 1.16 years – if the recent increase in mean BMI is projected forward (both estimates assuming a constant composition of mortality risks).

Predicted losses of longevity were somewhat more sensitive to assumptions about changes in the composition of mortality risks by 2015 – with the predicted course of diabetes risks holding the key. This is illustrated by the black sections of the bars in Figure 1 which show the increase in attributable deaths implied by an increased share of diabetes in overall mortality risks, as entailed in the WHO mortality projections. In Figure 1 these outweigh the effect of declining total (attributable and non-attributable) mortality risks from ischaemic heart disease.

Uncertainty over the future course of diabetes risk can be illustrated further with predictions for males aged 60 to 70. An extrapolation of recent trends in the proportion of deaths at this age assigned to diabetes would see the number of deaths from this cause in 2015 increase from 318 to 431 (in this age group). The WHO predictions, however, imply a much sharper increase – to 1318 deaths per year.

Under the least favourable scenario for 2015 – with continuing increases in mean BMI and a (not unrelated) sharp rise in the proportion of deaths from diabetes – the attributable loss of life expectancy is 1.28 years for both sexes combined.

## Discussion

Our base case results are broadly consistent with findings from other studies using similar methodology for the estimation of population attributable fractions. Our estimate of 41,000 attributable deaths (9% of all deaths) per year in England and Wales between 2003 and 2005 bears comparison with the National Audit Office's (NAO)[12] estimate of over 31,000 deaths per year attributable to "obesity" in England in 1998, when BMI levels were lower. Banegas et al. [13] estimated that 8.7% of all deaths in the UK were attributable to excess adiposity, which is very close to our own estimate. Using a similar methodology, Ni Mhurchu et al. [14] estimated 11% of all deaths in New Zealand for the year 1997 were attributable to excess adiposity. This amounted to 83% of all diabetes deaths, 24% of all ischaemic heart disease deaths and 15% of ischaemic stroke deaths. Ni Mhurchu et al. also found that higher-than optimal BMI was most prevalent in the 55–64 years old age group, as opposed to the slightly older age group of 60–69 years of age of our own estimates.

We have compared our cause-specific effect estimates with those of the Prospective Studies Collaboration (PSC) analyses of results from 900,000 subjects [15]. The increments of risk per unit of BMI that are employed here lie close to and on either side of the values in Table 2 of the PSC report. Exact comparison is precluded as the PSC slopes begin at BMI 25 kgm^-2^whereas the existing CRA slopes extend down to the counterfactual mean of BMI = 21 kgm^-2^. The comparability of the exposure risk slopes also addresses the issue that the CRA implementation (which we have employed) uses slopes of incidence on exposure which could, in principle, be biased as proxies for mortality on exposure. The PSC however reports on the metrics of mortality.

### Potential biases in the methods employed

There are a number of potential sources of bias in methods employed. Firstly, our method may yield biased estimates when used with relative risks adjusted for confounding. This occurs when adiposity is correlated with other exposures or unobserved factors in the population of interest, resulting in under- (when there is a positive correlation) or over- (negative correlation) estimation of the true PAF when used with adjusted relative risks [16-18]. However, the information requirements of confounder adjusted methods effectively preclude their use here. Flegal et al. have illustrated the potential magnitude of the biases associated with different analytic methods [18] and recommend the use of sensitivity analyses. Potential confounding by smoking has been a concern in this field, but the PSC analyses found virtually identical slopes of risk on exposure for never smokers and current smokers (Figure 6, [15]).

Secondly, the methods used here are based on risks observed in subjects mostly recruited and measured in middle age in the 1980s and 90s. The cohorts currently in middle and late middle age will typically have gained adiposity at an earlier age and this may confer risks additional to those considered here. Adiposity in adolescence appears to be strongly predictive of later risk [19,20], but studies that relate risk to measures of exposure integrated over the lifespan are apparently few.

In addition, causes other than those examined in our study may be positively associated with excess adiposity, which biases our estimates of attributable deaths downward.

### Predictions

The Foresight model predicted that the increase in adiposity between 2004 and 2015 would reduce life expectancy by about 0.11 years in men and 0.02 years in women (Figure 18 of their report)[1]. This is comparable with our own projection for this period, with reduction of 0.28 years (derived from the model). These estimates, based on very different methodologies, suggest relatively minor effects on life expectancy from rising adiposity. Much of the reason is that the leading cause of death whose risk is increased by adiposity (ischaemic heart disease) is projected, for other reasons, to decline as a proportion of total mortality risks. This assumption may not be robust.

We have sought to contain the uncertainty arising from interdependent trends in adiposity and related mortality risks by using the Government Actuary's predictions for 2015 to define an all cause mortality "envelope" within which the sum of specific causes of death is constrained to fit. This (arbitrarily) restrains uncertainty to the proportions of deaths likely to be from each cause. A defence of this approach is that official projections of all cause mortality, such as those of the Government Actuary, have an unbroken record of conservatism: things have (so far) invariably turned out better than expected [21]. There can, however, be no guarantee that that this will continue to be so in the future.

## Conclusion

Excess adiposity appears to contribute materially but modestly to mortality risks in England and Wales and this contribution is likely to increase in the future. The robustness of these estimates is limited by the lack of control for correlated risks by stratification and by the empirical uncertainty surrounding the effects of prolonged excess adiposity beginning in adolescence.

Work to reduce uncertainty in predictions like these needs to concentrate on the mutual relationships between evolving levels of adiposity and the composition of mortality risks in the short to medium term future. It is not easy to model such interdependent trajectories in Excel. Microsimulation methods, as used in the Foresight report, may have a useful role in modelling such trajectories. Cell-based methods like ours and microsimulation methods could thus continue to play complementary roles. Both will remain dependent on the quality of epidemiological data mapping these evolving epidemics.

## Competing interests

The authors declare that they have no competing interests.

## Authors' contributions

JP conceptualized the project, and contributed to the interpretation and writing of the manuscript. CK constructed the working computer model and contributed to the writing of the manuscript. SM was involved in obtaining datasets and projections for the model, and writing up the manuscript. NP supervised and contributed towards the entire process of model construction, and edited the manuscript. All authors have read and approved the final manuscript.

## Supplementary Material

Additional file 1 **Overview of procedure for estimating mortality attributable to excess adiposity**. Description of the computer model to estimate mortality attributable to excess adiposity. Click here for file

Additional file 2 **WHO Comparative Risk Assessment methodology – implementation in Excel**. Explanation of the Comparative Risk Assessment methodology in estimating attributable mortality due to continuously distributed risk factors. Click here for file

Additional file 3 **Computer model: estimated and projected mortality attributable to excess adiposity in England and Wales for the years 2003–2015**. Computer model to estimate mortality, years of life lost (YLL, under varying weighting assumptions) and loss of life expectancy attributable to excess adiposity. Click here for file
