# Supplementary material for: Mortality attributable to excess adiposity in England and Wales in 2003 and 2015: explorations with a spreadsheet implementation of the Comparative Risk Assessment methodology
Source: Popul Health Metr. 2009 Jun 30;7:11. doi: 10.1186/1478-7954-7-11 (PMC2714074; doi:10.1186/1478-7954-7-11)
Supplement: Additional file 1 — Overview of procedure for estimating mortality attributable to excess adiposity. Description of the computer model to estimate mortality attributable to excess adiposity. [file 1478-7954-7-11-S1.doc]

**Additional file 1**

**Overview of procedure for estimating mortality attributable to excess adiposity**

Kelly C et al, 2009

From a range of specific inputs, many of which are user-selectable, the model estimates deaths, years of life lost and life expectancy lost attributable to higher than optimal BMI.

The structure of the model is demonstrated in Error: Reference source not found.1. Inputs are flagged by their blue boxes (solid border), processes/ analysis are shown by their green boxes (dashed border) and outputs are presented in orange boxes (dotted border).

The model is structured into sheets for each individual process, working from right to left in the Excel tabs. All workings are fully dynamic; if the source data is altered, the final outputs (both tables and graphs) will adjust accordingly.

All set-up options are located on a single sheet, and these options drive the rest of the workbook.

Sensitivity analyses are performed by macro-driven calculations, altering the model and generating sensitivity tables based upon each scenario. These are calculated on-demand when desired.

**Figure A.1.1: Structure of model for estimating mortality attributable to excess adiposity**

**Historical (observed)**

**BMI (mean, sd)**

*Source: HSE 1997-2004*

*-*

**Population**

*Historical / official forecasts (ONS)*

**Relationship (slope) of**

**logRR on BMI**

by cause of death, sex

and age (James et al, 2004)

**Counterfactual BMI**

**Distribution**

Mean / standard deviation

*Source: WHO GBD Study*

**Life Table a_x_ values**

**Attributable Years of Life Lost (YLL)**

by cause of death, age, sex

**Attributable Deaths**

by cause of death, age, sex

(relative to counterfactual)

**Multiple Decrement Life Tables**

Based upon methods illustrated

by Preston,

Heuveline

,

Guillot

2001

**BMI Forecasts**

Variable future scenarios, based on linear

regression, weighted to population size

*3 different scenarios*

**Mortality Forecasts**

by cause of death, age, sex

**WHO Comparative Risk**

**Assessment Method**

Quantifying burden of disease due to

specified risk factors

**Attributable loss of Life**

**expectancy**

*(Human Mortality*

*Database)*

**All cause mortality
projections***(GAD)*

**Composition of mortality***(historical, ONS)*

**Projected composition of mortality***(No change, WHO) ONS)*

**Key:** Blue (solid border) = inputs; green (dashed border) = processes/analysis; orange (dotted border) = outputs.
