# Supplementary material for: Mortality attributable to excess adiposity in England and Wales in 2003 and 2015: explorations with a spreadsheet implementation of the Comparative Risk Assessment methodology
Source: Popul Health Metr. 2009 Jun 30;7:11. doi: 10.1186/1478-7954-7-11 (PMC2714074; doi:10.1186/1478-7954-7-11)
Supplement: Additional file 2 — WHO Comparative Risk Assessment methodology – implementation in Excel. Explanation of the Comparative Risk Assessment methodology in estimating attributable mortality due to continuously distributed risk factors. [file 1478-7954-7-11-S2.doc]

**Additional file 2**

**WHO Comparative Risk Assessment Methodology – Implementation in Excel**

Kelly et al, 2009

The WHO Comparative Risk Assessment (CRA) project estimated global disease burdens attributable to leading risk factors in 2000. To deal appropriately with continuously distributed risk factors such as blood pressure and BMI a new methodology was implemented [1][2]. We have adapted this methodology (as supplied by the Comparative Risk Assessment investigators) and are providing it here in order to facilitate its wider use.

Conceptually, the Population Attributable Fraction is the fraction by which the occurrence of a disease of interest would be reduced under an alternative, more favourable, exposure distribution. For assessing the full effects of a given exposure distribution the appropriate comparator (or counterfactual) is a distribution deemed likely to confer ‘theoretical minimum risk’.

$$\begin{matrix} \text{PAF}=\frac{\text{attributable cases}}{\text{total cases}} \\ =\frac{\text{factual cases - counterfactual cases}}{\text{factual cases}} \end{matrix}$$

These aggregates of cases (risk) can be thought of a proportional to ‘areas under the curve’, that is as integrals of ‘proportion at a given level of exposure’ x ‘risk at that level of exposure’, in other words as:

$$=\frac{\int_{\text{x}=0}^{\text{m}} \text{RR(x)P(x)dx}-\int_{\text{x}=0}^{\text{m}} \text{RR(x)P'(x)dx}}{\int_{\text{x}=0}^{\text{m}} \text{RR(x)P(x)dx}}$$

Where P’ is the proportion at the given exposure level under the counterfactual distribution and m is the maximum level of exposure.

If the continuous distributions are envisaged as made up of multiple very small strata, then an equivalent, discrete, form of the equation is:

$$\begin{matrix} \text{PAF}=\frac{\sum\text{p}_{\text{1i}}\text{RR}_{\text{i}}-\sum\text{p}_{\text{2i}}\text{RR}_{\text{i}}}{\sum\text{p}_{\text{1i}}\text{RR}_{\text{i}}} \\ \end{matrix}$$

where p_1_ refers to the factual and p_2_ to the counterfactual distribution. Given that, in the method adopted here, the RRs for all positions on the counterfactual are set to 1 and the sum of the probability distribution is 1, this simplifies to:

$\text{PAF}=\frac{\sum\text{p}_{\text{1i}}\text{RR}_{\text{i}}-1}{\sum\text{p}_{\text{1i}}\text{RR}_{\text{i}}}$ (Equation used in calculations)

This discrete form can be conveniently implemented in Excel.

A graphical representation is given in Figure 1. The population of interest (or study population) has an exposure distribution with a mean of *x^—^*and a standard deviation of *s*, compared to the ‘theoretical minimum risk’ (counterfactual) distribution with a mean of $\overline{\mu}$ and standard deviation of σ.

A given random member of the population of interest may be taken to have a BMI of x. Under the counterfactual this individual is assumed to occupy a similar position relative to the mean –expressed in sd units, or z-scores – in the counterfactual distribution. This constancy of relative position in the distribution as the overall level of exposure becomes more favourable might be thought of as resulting from genetic propensities: those with a genetic propensity to thinness would stay on the ‘thin’ side of the mean. Given that the aim of comparative risk assessment is to quantify the effects of alternative environmental exposure distributions this ‘controlling out’ of intrinsic individual differences is both desirable and convenient.

**Figure 1:** CRA methodology – comparison of population of interest with actual (factual) exposure distribution and with a corresponding theoretical optimum distribution (the counterfactual)

**Key***: x^—^* *=* mean BMI for population of interest; *s* = standard deviation for population of interest; $\mu^{-}$ *=* mean BMI for counterfactual distribution; $\sigma$ = standard deviation for counterfactual distribution; *x* represents a random individual in the population of interest, relocated to the equivalent position in the counterfactual distribution to assess their risk of mortality at the new

The lower part of Figure 1 shows the distance (in BMI units) that random subject x travels on the exposure axis in moving to his or her corresponding position on the counterfactual distribution. This distance times the slope of log relative risk on BMI gives the proportional reduction in risk (i.e. the distance travelled on the ‘log RR’ axis), equivalent to the potential impact fraction for this individual. The slope of log RR on BMI was obtained by meta-analysis for the Comparative Risk Assessment [1]. The assumed log-linear relationship between exposure levels and risk has been shown to be a good approximation for many of the leading ‘nutritional-physiological’ group of risk factors, including BMI [1].

This approach has been implemented in Excel. The population distribution of BMI by age/sex groups is represented in the body of each spreadsheet with one row for each small stratum – with a possible range from BMI=10.0 at row 17 to BMI=60.0 at row 117 (only part of this range is actually used). Each active cell in the range F17 to U117 estimates the $\text{p}_{\text{1i}}\text{RR}_{\text{i}}$component of the PAF equation above for the age/sex category represented by the column – by a procedure explained below.

These elements are then summed across all the strata ($\sum\text{p}_{\text{1i}}\text{RR}_{\text{i}}$) to evaluate the overall PAF (using the formula above). These resulting PAFs are shown in row 10.

**Procedure for estimating $\text{p}_{\text{1i}}\text{RR}_{\text{i}}$for each i (rows 17 to 117).**

The formula as implemented in Excel (with cell references corresponding to those in *Kelly_et_al_mortality_attributable_to_excess_adiposity_excel_model.xls* ) for the BMI stratum 17.0 to 17.5 (row 31) and age/sex group M45 (males 45-60) is:

=(NORMDIST($B31,Setup!J$27,Setup!J$28,FALSE)/(1/0.5))*MAX(1,EXP(($B31-((($B31-Setup!J$27)/Setup!J$28)*Setup!AE$28+Setup!AE$27))*J$14))

which can be presented in a more manageable format in the following equation:

$$\frac{\text{NORMDIST}\left(\text{X, }\overline{x}\text{, }s\right)}{\frac{1}{\text{Interval width}}}\times\text{MAX}\left(\text{1,e}^{\left(\text{X }-\text{ }\left[\frac{\text{X}-\overline{x}}{s}\times\sigma_{\text{c}}\right]+\mu_{\text{c}}\right)\times\text{log RR }}\right)$$

**Key***: x^—^* *=* mean BMI in the population of interest; *s* = corresponding standard deviation; $\mu^{-}$ *=* mean BMI in chosen counterfactual distribution; $\sigma$ = corresponding standard deviation; *x* represents a random individual in the population of interest, relocated to the equivalent position in the counterfactual distribution to assess their relative risk of mortality at the new exposure level.

The first part of the equation (before the multiplication sign) estimates the proportion of the BMI distribution in the population of interest that lies in the small stratum of interest, i ($\text{p}_{\text{1i}}$) using the Excel NORMDIST function – given the mean and standard deviation of the distribution as supplied in the 2^nd^ and 3^rd^ arguments. (The fourth argument – not shown – is set to ‘false’ to indicate that a cumulative distribution is not required.)

The sum of the proportions of the distribution (using NORMDIST) are set to total 1 when the interval width for x is 1. Here an interval width of 0.5 BMI units is used so the value of each proportion is divided by 2 (1/interval width) to preserve a sum of 1.

The second part of the equation evaluates RR_i_, i.e. the risk of the outcome of interest for that stratum relative to the corresponding position on the counterfactual distribution.

| $\text{MAX}\left(\text{1,e}^{\left(\text{X }-\text{ }\left[\frac{\text{X}-\overline{x}}{s}\times\sigma_{\text{c}}\right]+\mu_{\text{c}}\right)\times\text{log RR }}\right)$ |  |  |
| --- | --- | --- |
| **Considering the formula in its individual constituents:** | | |
| $\text{MAX}\left(\text{1,e}^{\left(\text{X }-\text{ }\left[\frac{\text{X}-\overline{x}}{s}\times\sigma_{\text{c}}\right]+\mu_{\text{c}}\right)\times\text{log RR }}\right)$ |  | The highlighted portion evaluates x’s distance from the mean of the factual distribution (in the population of interest) in sd units (i.e. the z-score) |
| $\text{MAX}\left(\text{1,e}^{\left(\text{X }-\text{ }\left[\frac{\text{X}-\overline{x}}{s}\times\sigma_{\text{c}}\right]+\mu_{\text{c}}\right)\times\text{log RR }}\right)$ |  | The highlighted elements show how the z-score for the factual distribution is applied using the mean and standard deviation of the counterfactual distribution to find the position (BMI value) on the counterfactual distribution that corresponds to the position on the factual distribution (assuming symmetrical normality). |
| $\text{MAX}\left(\text{1,e}^{\left(\text{X }-\text{ }\left[\frac{\text{X}-\overline{x}}{s}\times\sigma_{\text{c}}\right]+\mu_{\text{c}}\right)\times\text{log RR }}\right)$ |  | Subtracting the position on the counterfactual from the position on the factual gives the difference in exposure (in BMI units) |
| $\text{MAX}\left(\text{1,e}^{\left(\text{X }-\text{ }\left[\frac{\text{X}-\overline{x}}{s}\times\sigma_{\text{c}}\right]+\mu_{\text{c}}\right)\times\text{log RR }}\right)$ |  | LogRR here stands for the slope i.e. change in logRR per 1 unit change in BMI. These values are taken from the over-views performed for the Comparative Risk Assessment.[[1]](#endnote-2) Multiplying the 2 together gives the change in risk on a log scale. |
| $\text{MAX}\left(\text{1,e}^{\left(\text{X }-\text{ }\left[\frac{\text{X}-\overline{x}}{s}\times\sigma_{\text{c}}\right]+\mu_{\text{c}}\right)\times\text{log RR }}\right)$ |  | Exponentiating the change in risk on a log scale gives the change in risk on an arithmetic scale. Because the risk on the counterfactual distribution is set to 1 (for all positions on that distribution), this now equals the RR for the corresponding BMI value on the actual distribution. |
| $\text{MAX}\left(\text{1,e}^{\left(\text{X }-\text{ }\left[\frac{\text{X}-\overline{x}}{s}\times\sigma_{\text{c}}\right]+\mu_{\text{c}}\right)\times\text{log RR }}\right)$ |  | If the value of the exponential part of the formula returns a result smaller than 1 (which should not happen), 1 is the default value to be returned instead. In other words all values on the counterfactual distribution are required to be more favourable than the corresponding values on the factual distribution. |

The summed products of the proportions in exposure stratum i and the relative risk at this exposure level ($\sum\text{p}_{\text{1i}}\text{RR}_{\text{i}}$) yields the Population Attributable Fraction via the equation:

$$\text{PAF}=\frac{\sum\text{p}_{\text{1i}}\text{RR}_{\text{i}}-1}{\sum\text{p}_{\text{1i}}\text{RR}_{\text{i}}}$$

**Advantage of the CRA Methodology**

In contrast to the conventional approach of dividing BMI into ‘normal’, ‘overweight’ and ‘obese’ (or just ‘normal’ and ‘obese’) categories, exposure to excess adiposity has been modelled using a method which more closely approximates the underlying continuous distribution [3]. The categorical approach, illustrated in its simplest (binary) form in Figure 2, arbitrarily divides the population of interest into ‘normal’ and ‘obese’ portions using the BMI cut point of 30. For a binary adverse risk factor the conventional formula is:

$$\begin{matrix} \text{PAF}=\frac{\text{attributable cases}}{\text{total cases}} \\ =\frac{\text{p(RR}-\text{1)}}{1+\text{p(RR}-\text{1)}} \end{matrix}$$

Applied to adiposity, this method assigns a common relative risk level to all with BMIs below 30 (set to 1) and to all above 30 (the relative risk for ‘obesity’).

**Figure 2:** Treatment of adiposity as a binary exposure, with 2 levels ‘normal’ and ‘obese’

There are two major shortcomings of the categorical approach when applied to continuous exposure distributions:

1. Relative risks may be biased because of potential heterogeneity in exposure distributions within both the ‘normal’ and ‘obese’ categories in the studies contributing to the meta-analyses - from which the summary Relative Risks are obtained.
2. The meaning of the ‘Population Attributable Fraction’ is fundamentally unclear because the implicit counterfactual distribution (a population with its BMI distribution simply truncated at some threshold (here at 30)) is seriously unrealistic. This means that the comparison state under which the burden of the diseases of interest would be reduced cannot be clearly imagined.

**Limitation of the CRA methodology**

The CRA methodology has its own limitations:

- **It assumes a symmetrical normality.** The only input parameters are mean / standard deviation, with no possibility of defining skew. As exposures change within a population, the distribution may change subtly in its skew – i.e. with BMI, as average BMI increases, it may do so with a positive skew. The fact that the model does not take account for this means that underestimation/overestimation is possible, depending on the exposure type in question.
- **Mathematical approximation to the continuous formula.** While the approximation is close, it will never be perfect unless calculus is implemented. Interval widths are set at 0.5 BMI units, which is likely to provide a close approximation.

**References**

1.Ezzati M, Lopez AD, Rodgers A, Murray CJL. eds *Comparative quantification of health risks: global and regional burden of diseases attributable to selected major risk factors.* Geneva: World Health Organization; 2004. (Vol 1,2 3 is CDROM).

2. Hoorn SV, Ezzati M, Rodgers A, Lopez AD, Murray CJL. **Estimating attributable burden of disease from exposure and hazard data**. In: Ezzati M, Lopez AD, Rodgers A, Murray CJL, editors. *Comparative quantification of health risks: global and regional burden of diseases attributable to selected major risk factors, vol 2.* Geneva: World Health Organization; 2004; p 2129-40.

3. James WPT, Jackson-Leach R, Mhurchu CN, Kalamara E, Shayeghi M, Rigby NJ, Nishida C, Rodgers A. **Overweight and obesity (high body mass index)**. In: Ezzati M, Lopez AD, Rodgers A, Murray CJL, editors. *Comparative quantification of health risks: global and regional burden of diseases attributable to selected major risk factors, vol 1.* Geneva: World Health Organization; 2004; p. 497-596.

1. [↑](#endnote-ref-2)
